# Research progress on adsorption and separation of carbonyl sulfide in blast furnace gas

**DOI:** 10.1039/d2ra07409e

**Published:** 2023-04-24

**Authors:** Ying Wang, Xiaoqin Wu, Di Wei, Yue Chen, Jia Yang, LvYou Wu

**Affiliations:** a Key Laboratory of Hubei Province for Coal Conversion and New Carbon Materials, School of Chemistry and Chemical Engineering, Wuhan University of Science and Technology China wuxiaoqin@wust.edu.cn

## Abstract

The iron and steel industry is one of the foundational industries in China. However, with the introduction of energy-saving and emission reduction policies, desulfurization of blast furnace gas (BFG) is also necessary for further sulfur control in the iron and steel industry. Carbonyl sulfide (COS) has become a significant and difficult issue in the BFG treatment due to its unique physical and chemical properties. The sources of COS in BFG are reviewed, and the commonly used removal methods for COS are summarized, including the types of adsorbents commonly used in adsorption methods and the adsorption mechanism of COS. The adsorption method is simple in operation, economical, and rich in types of adsorbents and has become a major focus of current research. At the same time, commonly used adsorbent materials such as activated carbon, molecular sieves, metal–organic frameworks (MOFs), and layered hydroxide adsorbents (LDHs) are introduced. The three mechanisms of adsorption including π-complexation, acid–base interaction, and metal–sulfur interaction provide useful information for the subsequent development of BFG desulfurization technology.

## Abstract 2

Carbonyl sulfide (COS) causes issues with blast furnace gas (BFG) treatment due to its unique physical and chemical properties. Here, we review the sources of COS in BFG and summarise the most common COS removal methods, including the most common types of adsorbents.

## Graphical abstract

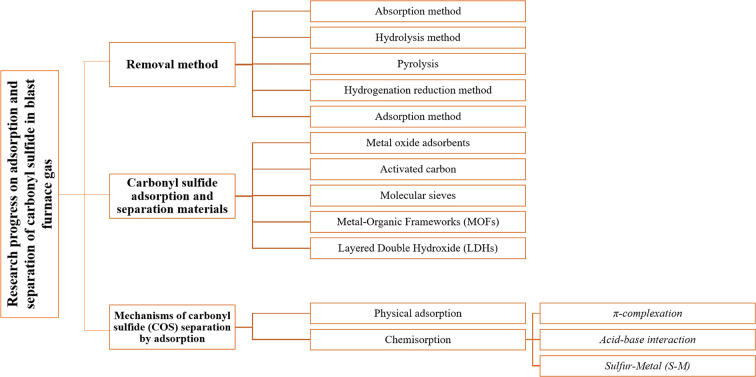

The iron and steel industry is an important part of Chinese economy. The output of iron and steel has been ranked first in the world for 24 consecutive years, which reached up to 877 million tons in 2021.^1,2^ As a traditional basic industry, large amounts of materials and energy are used in the production process, and there are many engineering and environmental problems that need to be solved urgently. One of the problems is the emission control of gas pollutants such as sulfur oxides and nitrogen oxides.^3,4^ Therefore, it is necessary to implement ultra-low emission policy in the iron and steel industry for further achieving environmental protection goals and serving ecological civilization strategies.^5,6^

Iron and steel enterprises are not only consumers of materials and energy but also producers of secondary energy, such as coke oven gas (COG), blast furnace gas (BFG) and converter gas (LDG), which are also important energy sources and raw materials. The green and efficient utilization of these gases is of great significance to the “two-carbon” goal and environmental protection. In recent years, the application of BFG combustion has become a new point of SO_2_ emission reduction. Analysis shows that it is mainly derived from the organic sulfur components in BFG. Therefore, in order to effectively control SO_2_ emission from BFG, source reduction of sulfur in BFG is imperative.

## 1 BFG desulfurization application and research trends

**Fig. 1:**
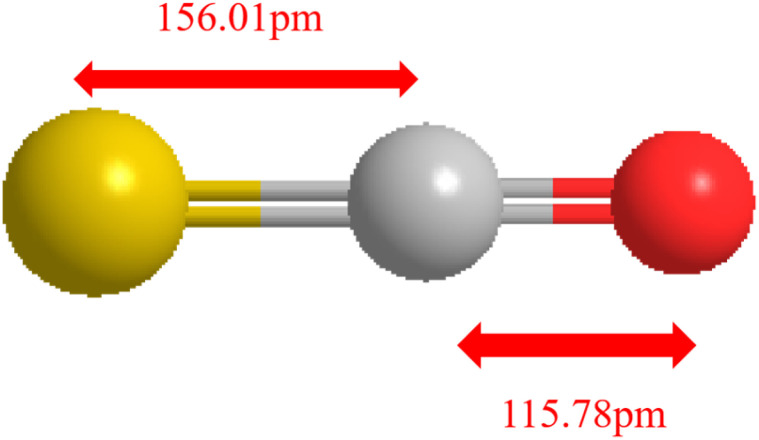
Chemical structure of carbonyl sulfide.

The main sulfur-containing compound in BFG is organic sulfur, accounting for more than 80% of the sulfur-containing components.^7–9^ The main components are carbonyl sulfide (COS), carbon disulfide (CS_2_), thiophene sulfur, *etc.* Some of the physicochemical properties of COS are shown in Table 1, and Fig. 1 is the schematic of the molecular structure of COS. COS is weak in acidity, small in polarity, and difficult to be separated. Limited by technical conditions and economic costs, COS has become a difficult point in BFG desulfurization.

**Table 1:** Physical properties of COS

Project	COS
Relative molecular mass	60.07
Melting point (°C)	−138.2
Boiling point (°C)	−50.2
Molecular structure	Straight
Bond length (pm)	116 (C–O); 156 (C–S)
Bond angle (°)	180
Key energy (kJ mol^−1^)	299.5 (OC–S); 593.3 (O–CS)
Dipole moment (D)	0.72

Presently, the technical routes of removing COS can be divided into two categories: one is the wet method and the other is the dry method. The wet method is relatively mature in industrial applications. Generally, liquids such as aqueous solutions or lye solutions (MDEA method, MSQ method, *etc.*) are used to absorb sulfur-containing gases.^10,11^ COS is converted into elemental sulfur for recycling. However, there are problems such as easy corrosion of equipment, difficult waste liquid treatment, and high operating costs in the wet method. Typical dry methods include hydrogenation reduction method, hydrolysis method, and adsorption method,^12,13^ which have the advantages of strong applicability, no corrosion, and simple operation but high technical requirements for operation, which limits the widespread promotion in the market. With the development of adsorption method, researchers have found that trace amounts of sulfur can deactivate the adsorbent and affect the desulfurization performance.^12,14–16^ Experimental results had proved that 4.00 mg of sulfur could reduce the activity of 1.00 g adsorbent Fe–Cu–K by about half.^5,6,17,18^ The crucial parameters for the adsorbent are selectivity, sulfur capacity, desulfurization strength, and stability, and further depth research on the performance indicators of comprehensive consumption should be carried out.

### 1.1 Absorption method

Absorption methods are commonly used in industries for large-scale industrial gas separation such as the separation of carbon dioxide (CO_2_) and hydrogen sulfide (H_2_S).^19^ It is also adequate to organic sulfur removal, and chemical absorption is more effective than physical absorption. Organic amine solutions such as monoethanolamide (MEA), diethanolamine (DEA), diethylene glycol amine (DGA), diisopropanolamine (DIPA), and methyl diethanolamine (MDEA) are usually selected as COS absorbents. Table 2 shows the kinetic study of COS reaction system with common absorbent solution. They can react with COS to generate corresponding thiamine salts for separation purposes, such as the alkyl alcohol amine method.^20^ A zwitterion mechanism could be used to describe the reaction of COS with primary amines (denoted here as AmH), which was similar to the absorption of carbon dioxide:^21^

1 COS + AmH → AmH + COS^−^

2 AmH + COS^−^ + B → AmCOS^−^ + BH^+^

**Table 2:** Kinetic study of COS-aqueous reaction system

Absorbent	Temperature/K	Solution/kmol m^−3^	Assumed rate constant/s^−1^	Quadratic rate constant/m^3^ kmol^−1^ s^−1^	References
MEA	298	1	16		21
MEA	298	1	15.1		22
DGA	300	5.7		2.3	23
DEA	298	1	11		21
MDEA	298	1.26–2.6		0.9	24

Rivera-Tinoco *et al.*^25^ studied the absorption of COS by dissolving DEA in methanol and modeled the process by a two-step method based on the zwitterion mechanism. However, in the process of alkanolamine desulfurization, part of the solvent will evaporate, the cost of the method is high, and by-products are easily produced, which will have impact on the environment.^26,27^ Second, the absorption method cannot reduce total sulfur (mass fraction) to less than 10^−6^ and is not suitable for the fine removal of COS. Therefore, it is necessary to develop catalysts with stable desulfurization performance to reduce process pollution and equipment loss, and improve the precision of COS drop rate.

### 1.2 Hydrolysis method

The hydrolysis method was originally used to remove H_2_S in the Claus process tail gas,^28^ and later it was gradually applied in the fields of industrial gas desulfurization and industrial tail gas treatment. The process mechanism is that COS reacts with H_2_O to generate H_2_S and CO_2_:

3 COS + H_2_O → H_2_S + CO_2_

The applicable temperature of the reaction is from 30 to 250 °C, and low temperature appears better for the reaction. Most of the hydrolysis catalysts play their role depending deeply on supports. The two common supports are metal oxides, such as γ-Al_2_O_3_, and non-metallic oxidants, such as activated carbon.^29,30^ Wang *et al.*^31^ explored the separation reaction of COS on Al_2_O_3_, and found that COS would preferentially undergo hydrolysis with water vapor and then undergo an oxidation reaction on an Al_2_O_3_ support. David *et al.*^32^ explored the reaction of COS and hydrogen cyanide (HCN) on titanium dioxide (TiO_2_) and found that there is a certain degree of competitive adsorption between the reaction sites of the two components. On this basis, by adjusting the pore size of the catalyst, it was found that the desulfurization performance can be effectively improved by increasing the specific surface area and surface basicity of the catalyst. Liang *et al.*^33^ synthesized ordered mesoporous carbons with basic sites by self-assembly, which could completely convert COS into H_2_S, and the abundant basic sites on mesoporous carbons could react with H_2_S to successfully convert COS into oxygen. Kan *et al.*^34^ also successfully separated COS by preparing organic carbon-containing basic active sites. Table 3 shows the hydrolysis of COS on different adsorbents.

**Table 3:** Hydrolysis of COS on different catalysts

Catalyst	Modification method	Active site	References
2D-NHPC^a^	Nitrogen doping	Pyrrole nitrogen	34
HTLCs^b^	Adjusting the Al/Ce ratio	–OH	35
Fe/MCSAC^c^	NH_3_	–NH_2_	36
NPC^d^	Nitrogen doping	Pyridine/pyrrole nitrogen	37
CuO/Fe_2_O_3_	Metal oxide	O–S/Fe–C	38
Mg/Al/Ce composite oxide	Metal oxide	M–OH	39
Graphene	Import C	Parallel graphene C–C sites	40
Cu–Fe/TSAC^e^	Metal oxide, O_2_	CuO, O_2_	41
Fe_2_O_3_/AC^f^	Metal oxide	Fe_2_O_3_	42

a 2D-NHPC: carbonization of urea and glucose to synthesize graphitized two-dimensional micro–meso–macroporous carbon

b HTLCs: Mg/Al/Ce-like hydrotalcite-like compounds.

c Fe/MCSAC: Fe-iron-based microwave coconut shell activated carbon.

d NPC: nitrogen-doped porous carbon.

e Cu–Fe/TSAC: preparation of Tobacco Stem Activated Carbon (TSAC) catalysts containing copper oxide and Fe_2_O_3_ by sol–gel method.

f Fe_2_O_3_/AC: activated carbon loaded with Fe_2_O_3_.

**Fig. 2:**
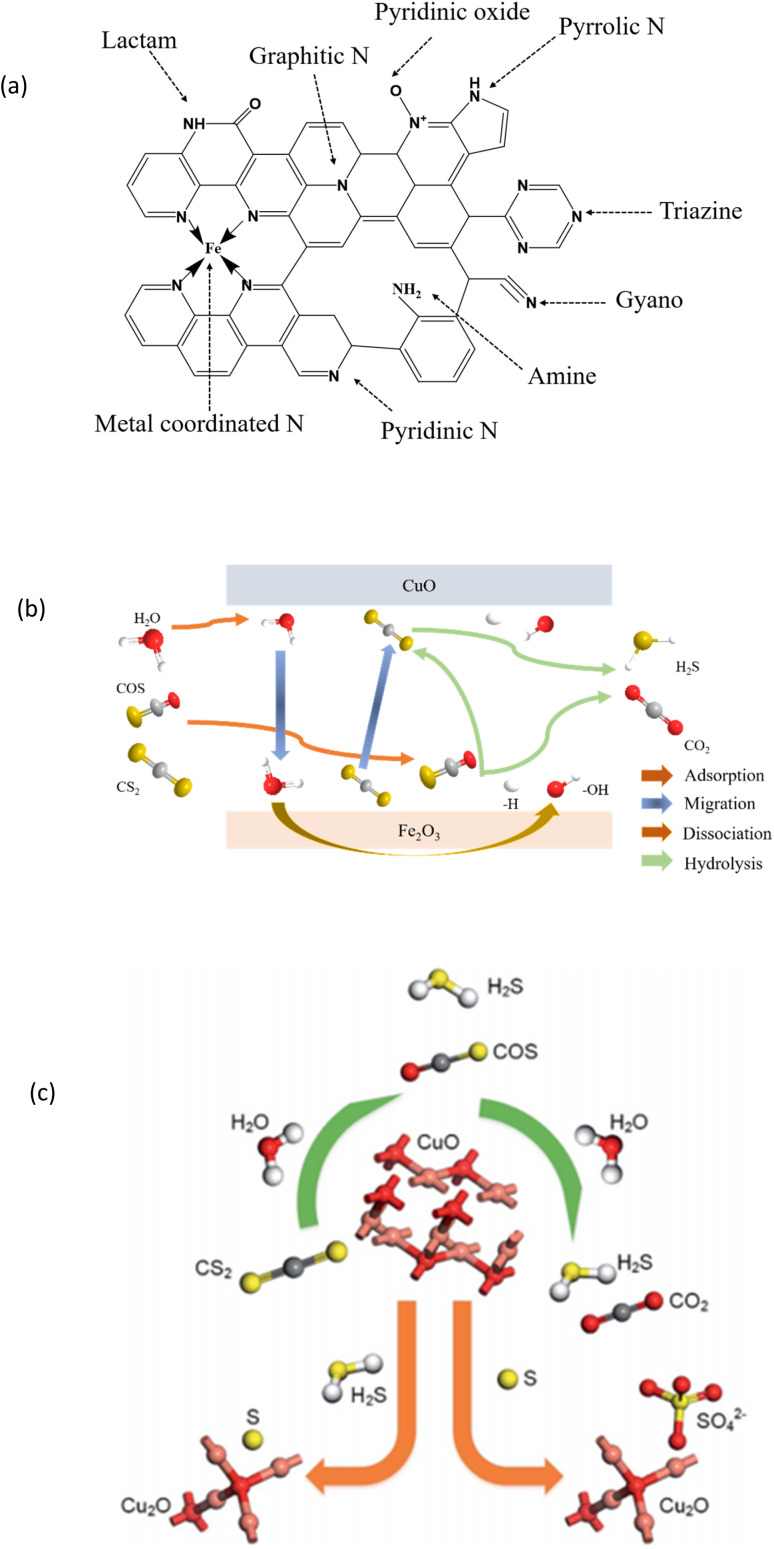
(a) Different types of nitrogenous sites in NPC (this figure has been adapted/reproduced from ref. 37 with permission from Royal Society of Chemistry, copyright 2016). (b) Hydrolysis mechanism of COS and CS_2_ on CuO/Fe_2_O_3_ catalysts. (c) Reaction process diagram of COS and CS_2_ on CuO (this figure has been adapted/reproduced from ref. 41 with permission from Royal Society of Chemistry, copyright 2018).

At present, it is generally believed that the hydrolysis reaction of COS is related to the basic sites on the surface of the catalyst, and hence, the hydrolysis capacity of the catalyst is often improved by increasing the alkalinity of the catalyst, such as increasing the number of nitrogen-containing bases and OH. Fig. 2(a) shows the various types of nitrogen functions observed in NPC materials.^37^ The most common types are pyridine nitrogen, pyrrolic nitrogen, and graphitic nitrogen. Other functions include amine, cyano, lactam, triazine, pyridine oxide, and metal coordination nitrogen. Sun *et al.*^41^ studied the reaction process diagram of COS and CS_2_ on Cu, as shown in Fig. 2(b) and (c).

After the hydrolysis catalyst reacts, a large amount of sulfur or sulfate will be deposited on the surface of the catalyst, blocking part of the pore size, reducing the specific surface area of the catalyst, and causing the catalyst to be poisoned and deactivated, and the desulfurization conversion rate will decrease. In addition, the reaction process is long, and it is easy to corrode the equipment, which will reduce the quality of the gas. The water vapor and CO_2_ generated by the reaction will undergo competitive adsorption with COS, increasing the difficulty of separation, so it is gradually replaced by other means.^43^

### 1.3 Pyrolysis

Pyrolysis is a method in which COS generates other substances *via* a thermal decomposition reaction. The reaction mechanism proposed by Partington *et al.*^44^ is now widely accepted. Under anaerobic or anoxic conditions, pyrolysis gases such as CO, H_2_ and CH_4_ are used to reduce sulfur-containing substances to produce simple sulfur through high temperature.^45^ In the presence of only carbonyl sulfur (COS), the thermal decomposition reaction is as follows:^46^

4 2COS → CS_2_ + CO_2_ (CO_2_ reaction)

5 COS → CO + S (CO reaction)

The study shows that the former is under the condition of hot metal platinum, and the latter is catalyzed by silica, in which the reaction of formula (5) is faster than that of formula (4), but the two reaction temperature windows are smaller, and the reverse reaction is very important for desulfurization. The impact of separation is not clear.^46^ Moreover, the utilization of pyrolysis method is limited because of low efficiency and high energy consumption.^44^

### 1.4 Hydrogenation reduction method

The hydrogenation reduction method refers to the method in which COS is converted into H_2_S *via* a hydrogenation reaction and then is removed. The main reaction is shown in formula (6) as follows:^17^

6 COS + H_2_ → CO + H_2_S

The reaction temperature is medium and high. Commonly used hydrogenation catalysts are binary or multi-element metals such as Co–Mo, Ni–Mo, Ni–W, and Fe–Mn. The main active components are transition metal elements, which are characterized by unfilled d-electron orbitals or hexagonal lattices, and there is a synergistic effect between the two metal elements to promote the hydrogenation reduction reaction. Wang *et al.*^47^ explored the hydro removal effect of Fe–Mn catalysts on COS, pointing out that COS is first converted into H_2_S in a reducing atmosphere, and the metal components on the catalyst promote its conversion into metal sulfides and water. Industrial catalysts are mostly prepared by impregnating metal components directly into a γ-Al_2_O_3_ support. Kamp E. *et al.*^48^ selected Pd/Al_2_O_3_ to completely convert COS into H_2_S at 200 °C, and the results showed that COS dissociated at the Pd site to form CO and S adsorbents, and COS would also adsorb onto the alumina support, thereby forming HSCO^2−^. Subsequently, HSCO^2−^ reacts with hydroxide surface groups to form carbon dioxide and hydrogen sulfide. In addition, molecular sieves with uniform and tunable mesopores and stable structures are also used as supports for hydrogenation catalysis. It has been studied that Ni–Mo and Co–Mo are supported on the surface of niobium-modified MCM-41, and dibenzothiophene can be separated by hydrogenation.^46^

The hydrogenation reaction can easily separate simple organic sulfur-containing compounds such as thiophene and its derivatives, heterocyclic sulfur compounds,^49^ but for the less polar COS, the reaction generates a new pollutant, H_2_S,^50^ which leads to equipment corrosion,^51,52^ increasing the running cost. At the same time, the hydrogenation method requires high reaction temperatures (temperature ≥ 400 °C, pressure ≥ 10 MPa), the use of hydrogen and precious metals, *etc.*, which require high equipment and control processes. Therefore, this method aims to achieve high removal rates under medium- and low-temperature conditions with low-cost catalysts.

### 1.5 Adsorption method

The adsorption method can separate one or several components simultaneously in gas using porous materials. Adsorption materials are metal oxides, activated carbon, molecular sieves, metal–organic framework (MOFs), layered double hydroxides (LDHs), *etc.*,^12,53–56^ which are characterized by stable and uniform framework structures and can be functionally selected according to separation requirements. Researchers often achieve geometric shape selection through the control of the microscopic morphology of materials. At the same time, metals and/or functional groups are used to modify the adsorption active site, change the electronic spatial structure of the microregion, and improve the adsorption selectivity *via* energy matching to meet the thermodynamic (BET three-constant adsorption isotherm) and kinetic requirements of the adsorption process. Zhao *et al.*^57^ prepared a series of nickel-containing hydrotalcite-derived oxides (HTOs) for the removal of COS by co-precipitation and found that Ni_3_Al–HTO had the best adsorption effect on COS. The study carried out ion exchange modification on NaX molecular sieves, and through DFT studies, it was concluded that COS achieved separation by forming S–M bonds with metal ions.^55^ The adsorption method has been favored by researchers due to its advantages of “plug and play” flexibility, easy operation and control.^55,58–61^ Therefore, the research progress and reaction mechanism of COS adsorbents are systematically described below.

## 2 Carbonyl sulfide adsorption and separation materials

### 2.1 Metal oxide adsorbents

Metal oxide adsorbents are mainly divided into iron-based, zinc-based, and aluminum-based oxide adsorbents. Due to the special physical and chemical properties of COS, COS is often converted into relatively unstable H_2_S for subsequent removal. Table 4 shows the different metal oxides.

**Table 4:** Removal of COS on different metal oxides

Metal oxide	Reaction formula	The main form	References
Iron base	Fe_2_O_3_·H_2_O + 3H_2_S → Fe_2_S_3_·H_2_O + 3H_2_O	γ-Fe_2_O_3_·H_2_O (maghemite)	62–64
	Fe_2_S_3_·H_2_O + 3H_2_S → 2FeS + S + 4H_2_O	α-Fe_2_O_3_·H_2_O (haematite)	
Zinc based	ZnO + COS → ZnS + CO_2_	ZnO	65 and 66
	ZnO + H_2_S → ZnS + H_2_O		
Manganese base	COS + H_2_ → H_2_S + CO	MnO	67
	COS + H_2_O → H_2_S + CO_2_		
	MnO + H_2_S → MnS + H_2_O		
Other	CuO + H_2_S → CuS + H_2_O	CuO	68–70

**Fig. 3:**
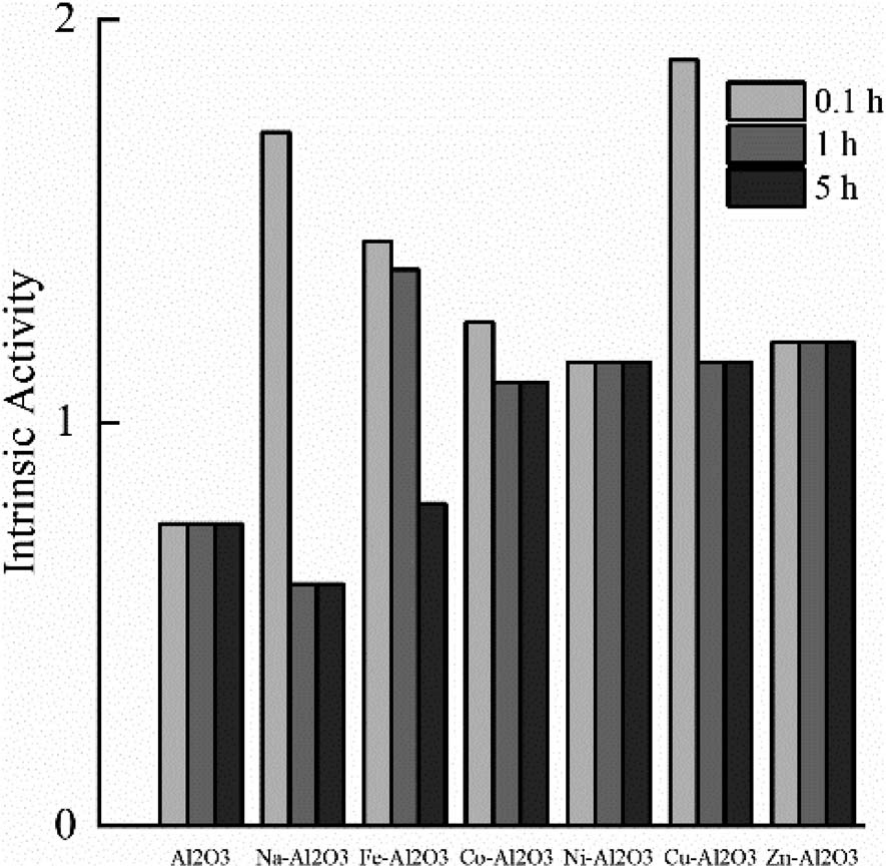
Reactivity (10^−6^ mol COS hydrolysis per m^2^ per h) as a result of reaction time (this figure has been adapted/reproduced from ref. 8 with permission from Elsevier, copyright 2012).

However, the desulfurization effect of single metal oxide adsorbents is not ideal, and hence, the desulfurization performance is often improved by adding unsaturated metal active sites *via* compounding metals. Jae *et al.*^71^ added rare earth metal Ce on the MgO support, which increased the sulfur adsorption capacity of the adsorbent by about 50 mg g^−1^ on the original basis. Yang *et al.*^29^ investigated the desulfurization performance of SnO_2_ doped with rare earth metal La. By comparing the saturated adsorption capacity of different La contents, it was found that with the increase in La loading, the pore size of the adsorbent became larger and the total pore volume increased. When the La content reaches 40%, the adsorbent can provide more catalytically active sites for contacting with COS, thereby improving the conversion rate of COS. Further research found that metal oxides such as iron-based and zinc-based cannot satisfy both high sulfur capacity and hydrothermal stability.^72,73^ In contrast, Al_2_O_3_ has good hydrothermal stability, and its excellent specific surface area can make active sites disperse uniformly, form a center-radical pore structure, reduce the diffusion resistance of the reaction, and improve the adsorption performance. As shown in Fig. 3, Rupp *et al.*^8^ improved the interaction between the catalyst and COS by supporting metal Pd on the γ-Al_2_O_3_ support. In order to improve the hydrolysis ability of COS at low temperatures, by loading transition metals (3 wt% Na, Fe, Co, Ni, Cu, Zn) on γ-Al_2_O_3_,^74^ it was found that the hydrolysis performance of the catalyst except for metallic Na doping was improved, indicating that metal modification could improve the initial and final activities of the hydrolysis reaction.

Both the hydrolysis and hydrogenation reduction reactions involving metal oxides produce H_2_S. With the progress of the reaction, the gas diffusion resistance to the solid surface increases, which increases the difficulty of desulfurization and reduces the desulfurization performance. Therefore, ensuring the activity of metal oxides, reducing the occurrence of side reactions, and improving the accessibility of reactions are the focus of later research.

### 2.2 Activated carbon

**Fig. 4:**
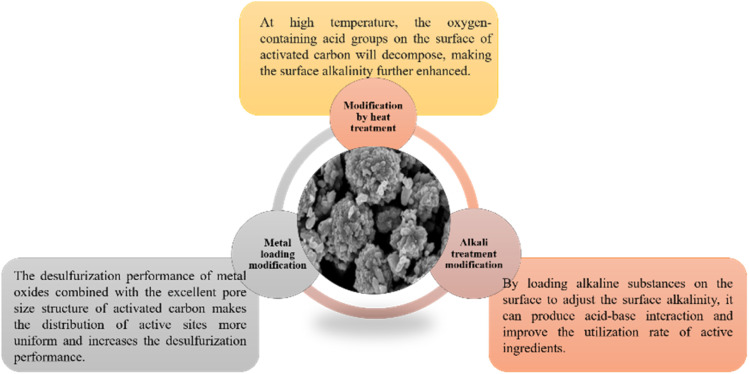
Different modification methods of activated carbon.

Activated carbon is an important adsorption material with abundant microporous structures^75^ and many functional groups on the surface. Its adsorption capacity can be increased *via* heat treatment modification, alkali treatment modification, and metal loading modification,^33^ as shown in Fig. 4. The activated carbon was modified with potassium salts, and the analysis found that potassium ions can be uniformly distributed on the activated carbon, which can improve the saturated sulfur capacity of the adsorbent.^75^ The Cu–Co–KW obtained by modifying the KOH-impregnated activated carbon with copper nitrate and sulfonated titanium cyan cobalt (CoPcs) has a saturated sulfur capacity as high as 43.30 mg COS per g. The improvement of adsorption performance is attributed to the combined effect of chemical adsorption and catalytic oxidation, among which chemical adsorption is the main one; in the reaction, an appropriate amount of water hydrolyzes part of COS, impregnation with KOH increases the basic functional groups on the surface of activated carbon, and the doped metal ions can accelerate the catalytic oxidation reaction with COS.^76,77^ Melanie *et al.*^78^ used activated carbon to adsorb COS and found that adding an appropriate amount of ammonia to the reaction mixture had a positive effect on the adsorption effect. However, activated carbon is mainly composed of micropores (about 95% or more), which play the role of selective adsorption, and its structure is easily collapsable and regeneration is difficult. In the process of industrial application, special attention should be paid to the activation temperature to avoid the effect of activated carbon aching on the desulfurization performance.

### 2.3 Molecular sieves

A molecular sieve is a general term for a class of crystalline aluminosilicate porous materials with a uniform pore size distribution and selective adsorption effect. Compared with the microporous structural characteristics of activated carbon, different pore sizes and cavities can be formed by connecting oxygen bridges to selectively prepare molecular sieves.^79–81^ Some studies have pointed out that the micropores in the molecular sieve provide the active sites for adsorption, and the mesopores increase the diffusion rate of the reaction.^82^

In addition to preparing molecular sieves with different pore sizes, the adsorption effect of molecular sieves can also be improved by surface modification. The cations on the molecular sieve framework can be replaced with other metal ions, to adjust the pore size, surface activity and other properties of the molecular sieve, and further improve the adsorption capacity and selectivity of the molecular sieve. The ion exchange adsorption of thiophene was carried out with metal Cu and Y molecular sieves. It was found that the adsorption capacity of the adsorbent was the best by self-reduction of Cu(ii)–Y to Cu(i)–Y. The adsorption form on the adsorbent was assumed, and it was concluded that the adsorption of thiophene was achieved by reacting with proton sites formed by reducing copper on Cu(i)–Y.^83^ At 40 °C, the breakthrough adsorption capacity of Ag/NaZSM-5 adsorbent for COS can reach 12.86 mg COS per g, and it shows good regeneration performance when dry air is used as the regeneration purge gas. The adsorption capacity is basically unchanged.^84^ Ryzhikov *et al.*^85^ selected different metal ions to modify the molecular sieve and found that the adsorption capacity of COS was in the following order: CaX ≈ BaX > NaX > CsNaX > NiNaX > NaY > CsY ≈ NiY. As shown in Table 5, the molecular sieves currently available for desulfurization mainly include: LTA-type molecular sieve, MFI-type molecular sieve and FAU-type molecular sieve.

**Table 5:** Characteristic structures of different molecular sieves

Molecular sieve type	LTA	MFI	FAU
Single cell chemical equation	Na_96_Al_96_·Si_96_·O_384_·216H_2_O	Na_*x*_Al_*x*_·Si_96−*x*_·O_192_·16H_2_O	Na_86_·Si_106_Al_86_O_384_·264H_2_O (Si/Al = 1.23)
Pore structure	Eight-membered ring	Two ten-membered ring cross structure	Twelve-membered ring
Maximum aperture	0.45 nm	0.63 nm	0.8–1.0 nm
Typical representative	Type A molecular sieve	ZSM-5 molecular sieve	X- and Y-type molecular sieves
Schematic	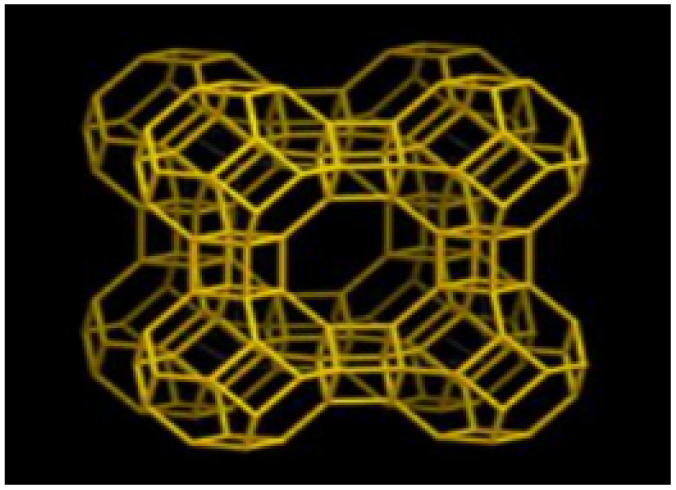	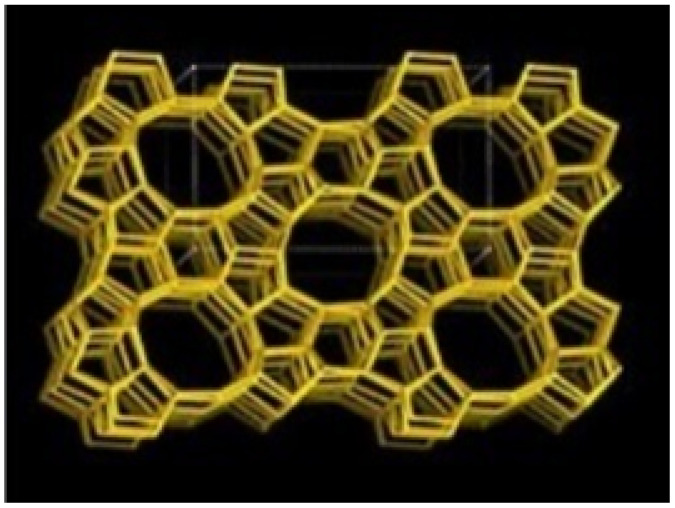	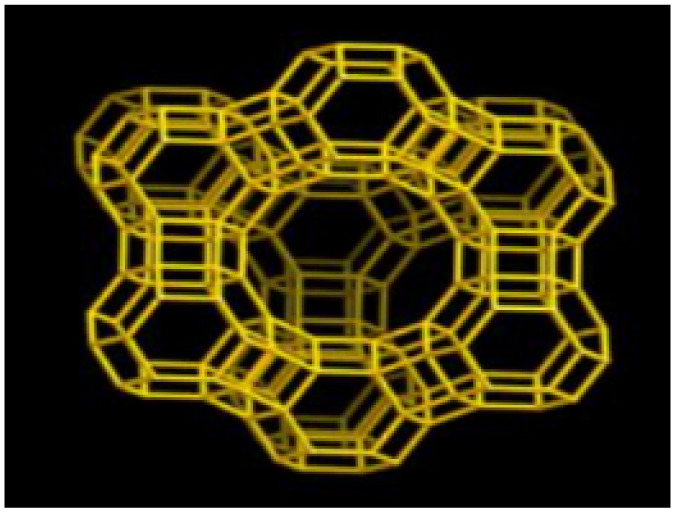

### 2.4 Metal–organic frameworks (MOFs)

MOFs are metal–organic framework materials composed of transition metal and organic ligand units self-assembled through coordination bonds. MOF materials have the same controllability of morphology, structure, and crystal size as molecular sieves, and the specific surface area of MOF materials is much larger than that of molecular sieves.^86–89^ They have become a popular adsorbent material in recent years. Britt *et al.*^86^ used a one-pot method to prepare metal–organic frameworks with different organic ligands, but some metal–organic frameworks have low hydrothermal stability and will compete with water during the adsorption process, resulting in the collapse of the adsorbent structure. The saturated adsorption capacity will decrease. Guo *et al.*^56^ synthesized MIL-53(Fe) by hydrothermal synthesis to hydrolyse COS and CS_2_, and found that the separation rate of COS was as high as 92.64%, and the 100% separation rate of CS_2_ could be maintained for about 30 min. By comparing the adsorption of SO_2_ on MOF-74 under different reaction conditions, it was found that the adsorption rate could reach 100% under dry conditions, but under wet conditions, the adsorption effect was reduced due to the competitive adsorption of water.^90^ However, due to the weak polarity of COS, it is still difficult to apply MOFs for separation, which is also the focus of further in-depth research.

### 2.5 Layered double hydroxides (LDHs)

Layered double hydroxides (LDHs), also known as hydrotalcite (HT) and hydrotalcite-like compounds (HTLCs), are a family of stacked compounds typically made of non-covalent inter layer anions and layer cations. Bond formation, the particle size and distribution of LDHs can be controlled by selectively regulating the types of anions and cations.^16^ The layered double metal hydroxide is weakly basic, but the double metal composite oxide (LDO) formed after calcination shows strong basicity and high specific surface area,^91^ and has abundant pore size and excellent catalytic performance and is widely used. The adsorption of COS on LDHs-derivative Ni/Mg/Al mixed oxide was 2.47 wt%, and the adsorption rate was almost unchanged after regeneration.^53^ The MA-LDHs-3 (Mg/Al = 3) adsorbent prepared by mechanical mixing has a large specific surface area and abundant OH-active sites. At the reaction temperature of 70 °C, the removal rate of COS reaches 100%, but the adsorbent will lose interlayer water at high temperatures, resulting in dehydration of hydroxyl groups on the laminate, resulting in skeleton collapse.^58^ Zhao *et al.*^92^ modified the hydrotalcite-derived oxide (HTO) with KOH, which not only increased the basicity of the adsorbent surface but also improved the Lewis basicity of Ni, making the adsorption and removal of COS the best. Although hydrotalcite is rich in basic sites, its specific surface area is small compared to other materials, and it is susceptible to structural collapse due to temperature effects, so the use of LDHs for desulfurization is not widely used.

## 3 Mechanisms of carbonyl sulfide (COS) separation by adsorption

COS adsorption is the process of being captured and enriched in the surface of the adsorbent, and there are physical and chemical processes, as well as the synergistic effect of the two processes. According to different adsorbents and adsorption reaction conditions, there are different COS adsorption reaction mechanisms.

### 3.1 Physical adsorption

**Fig. 5:**
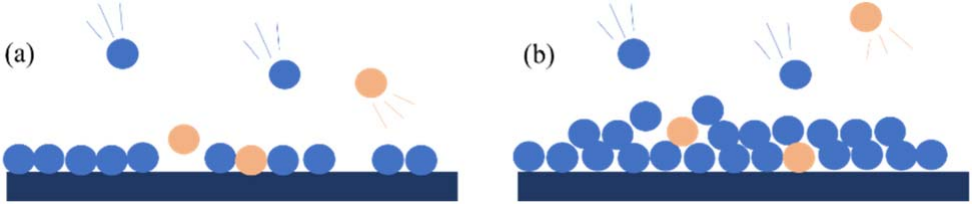
Simulated distribution of adsorbed gas on adsorbent surface. ((a) Shows simulated monolayer adsorption; (b) shows the multilayer adsorption results).

Physical adsorption is based on intermolecular forces (such as dispersion force, van der Waals force, and hydrogen bonding) to adsorb an adsorbate in the pores or surfaces of materials. Physical adsorption has almost no selectivity, and the adsorption is reversible. COS mainly occurs inside the micropores of the adsorbent, which requires the adsorbent to have a high adsorption capacity and strong selectivity. The Langmuir model is a classic model of physical adsorption that assumes that only a monolayer is formed on the molecular surface, as shown in Fig. 5(a). During the adsorption process, the gas molecules are in an equilibrium state of adsorption and desorption. But in the actual adsorption process, the surface of the adsorbent is not smooth and there is a complex pore structure. Based on the BET theory, it is believed that the physical adsorption of solid to gas is the result of van der Waals gravity, and there is also van der Waals force between molecules, so the adsorbent molecule may be adsorbed when it bumps into the adsorbed molecule, that is to say, the adsorption can form multi-molecular layers, and then multilayer adsorption and accumulation will be carried out on the adsorbent, as shown in Fig. 5(b).

**Fig. 6:**
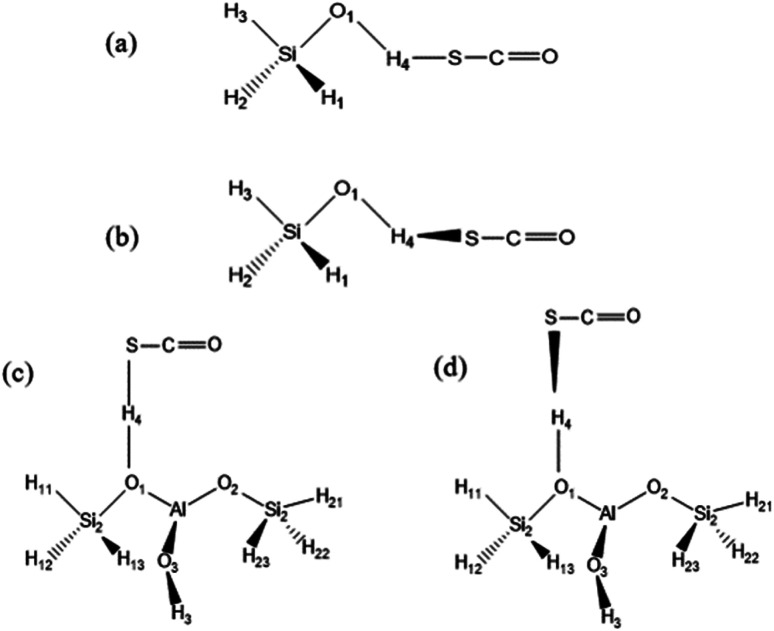
Four adsorption modes of COS on the silanol and bridging hydroxyl groups ((a) COS is adsorbed in parallel on the silanol; (b) COS is adsorbed vertically on the silanol; (c) COS is adsorbed in parallel on the bridging hydroxyl, and (d) COS is adsorbed vertically on the bridge hydroxyl).

In addition, the physical adsorption process is also controlled by the molecular sieve shape-selective adsorption mechanism. As the molecular sieve has a highly regular pore structure and a specific pore size, it has high selectivity for the size of the adsorbate molecules, but only substances whose molecular dynamics diameter is smaller than its pore size have adsorption capacity. For example, ZSM-5 molecular sieve, although its average pore size is about 0.55 nm, belongs to microporous zeolite, and hence, it can enter and interact with molecular sieves for COS molecules with a kinetic diameter of 0.53 nm. Based on the calculation of the electricity price rule, taking the fukalite molecular sieve as an example, the aluminum–oxygen octahedron AlO_2_(OH)_4_ and the silicon–oxygen tetrahedral structure in the framework make the molecular sieve structure layer display electroneutrality, and the aluminum–oxygen octahedron in the aluminum–oxygen octahedron is electrically neutral. About 60% of the Al–O bonds and Si–O bonds in the silicon–oxygen tetrahedron are ionic bonds and covalent bonds, respectively. Theoretically, the interlayer force of molecular sieves is the combination of hydrogen bonds and van der Waals forces between hydrogen and oxygen atoms. Therefore, the adsorption form of COS molecules into the molecular sieve adsorbent is shown in Fig. 6.

### 3.2 Chemisorption

Complexation, acid–base interaction, and S–M adsorption mechanisms can be divided according to the difference in active centers of COS action. Chemisorption is based on the principle of coordination chemistry, changing the surface acidity and alkalinity of the adsorbent and the distribution of active sites to improve the desulfurization performance.

#### 3.2.1 π-Complexation

**Fig. 7:**
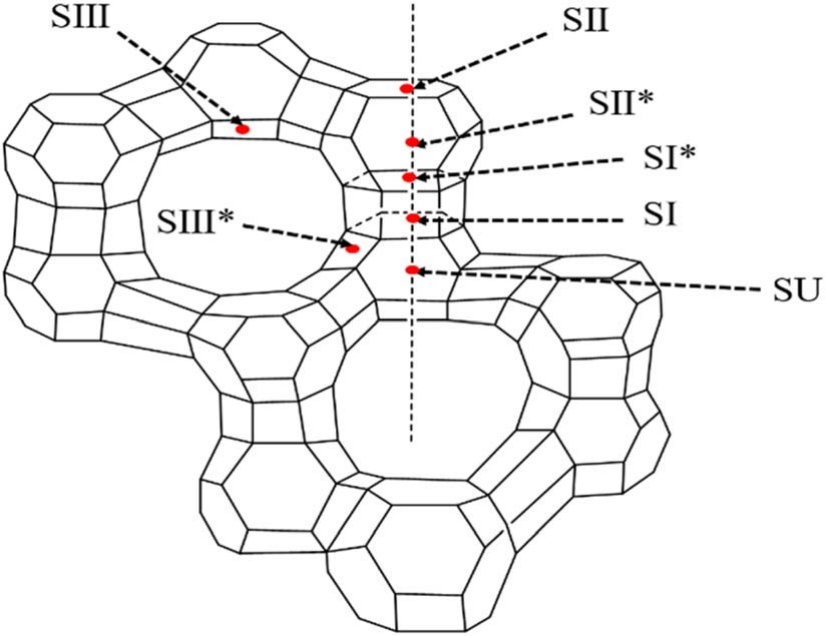
Cation sites on the FAU-type zeolite framework (this figure has been adapted/reproduced from ref. 95 with permission from American Chemical Society, copyright 1975).

Complexation is the formation of complex bonds between the adsorbate and the adsorbent to achieve adsorption and separation. In theory, all transition metal elements in the periodic table, that is, elements in the d block, can produce complex adsorption. When the transition metal atom has a ligand with greater electronegativity (such as F and Cl), due to the large shift of the electron cloud to the ligand, the metal will be partially positively charged, resulting in the outermost layer of the metal. When such metals come into contact with adsorbate molecules (*e.g.* CO and unsaturated hydrocarbons) with π electrons, they are easily receptive to the π electrons provided by the adsorbate to form σ bonds. At the same time, the metal feeds back the excess d electrons of the outer layer to the high-energy antibonding π* orbital of the adsorbed mass space, forming feedback π bonds. The synergistic effect of σ–π bonds enhances the bonding between metals and adsorbate molecules, and π-complex adsorption occurs.^93^ Palomino *et al.*^94^ used the gas-phase ion exchange (VPIE) method to modify Y molecular sieves, and the resulting Cu(i)–Y(VPIE) molecular sieves had the best terthiophene effect. The metal ion distribution of FAU-type molecular sieves is shown in Fig. 7. Due to the existence of coulombic repulsion, the occupancy of adjacent positions (such as SII and SII*) is rarely studied. It is assumed that the Cu(i)–Y(VPIE) zeolite has a Maxwell cation site distribution,^95^ so that Cu^+^ exposed at the SII and SIII sites is occupied by thiophene molecules, and the remaining Cu^+^ interacts with other molecules capable of -complexation (aromatic compounds and/or nitrogen heterocycles) for separation purposes.^96^

By analyzing the adsorption effect of Cu–Y on thiophene, it is concluded that some electrons on the p orbital of thiophene are donated to the empty s orbital of the metal (called an electron donor), and at the same time, the electrons on the d orbital of the metal donate electrons to the p* orbital of thiophene (called a p-donor). Molecular orbital theory further confirms the experimental results.^83^ In the case of complexation, the metal ions responsible for the adsorption process provide the transfer of electrons from the highest occupied molecular orbital (HOMO) to the lowest unoccupied molecular orbital (LUMO). Li *et al.*^97^ discovered that modifying activated carbon with metals such as Cu and Ag increased the amount of COS adsorbed by Cu(i). According to the research, the bond length of Cu(i)–S is shorter than that of Ag(i)–S, indicating that Cu(i)–S has a shorter bond length than that of Ag(i)–S. This is the reason that the -complex bond formed with COS is more stable.

#### 3.2.2 Acid–base interaction

The acid–base interaction is achieved by combining the Lewis base sites on the adsorbent surface with the acidic sulfur atoms of organic sulfides to achieve separation. Therefore, in order to increase the Lewis base sites on the surface of the adsorbent, metal salts are commonly used to modify the adsorbent, which are Fe, Cr, Al, *etc.* Song *et al.*^98^ combined DRTFIR analysis and found that the more the decrease in C–OH, the more S–O was generated during the catalytic process, so the C–OH group was conducive to the formation of S–O groups, among which –CH, –C–OH, and groups such as –C–OH and –COO play a huge role.

7 COS + C–OH → –CH + H_2_S

8 –COO + H_2_S → –CH + S–O

9 C–OH + H_2_S → –CH + S–O

COS removal process:

10 COS → H_2_S → S → SO_4_^2−^

**Fig. 8:**
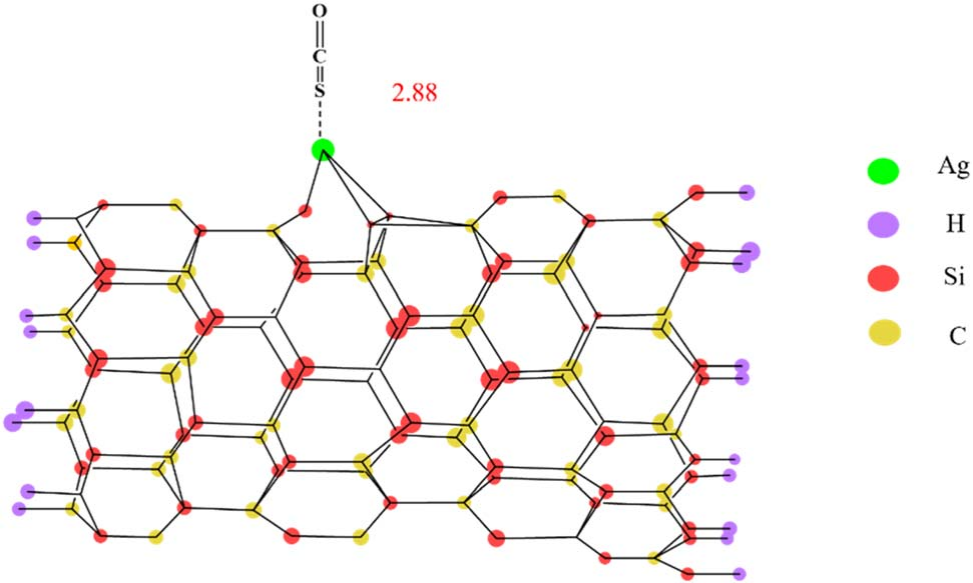
Stable adsorption model of COS on the Ag–SiCNT adsorbent (this figure has been adapted/reproduced from ref. 99 with permission from Springer, copyright 2015).

Peyghan *et al.*^99^ established the existence of Ag on SiCNT *via* DFT calculations. The analysis showed that the *E*_ad_ of the combined form in Fig. 8 is the largest (12.20 kcal mol^−1^). The study found that the hybridization of Ag atoms is close to sp^3^, which can provide more electron sites to COS, namely “Lewis's base” to improve the desulfurization performance.

**Fig. 9:**
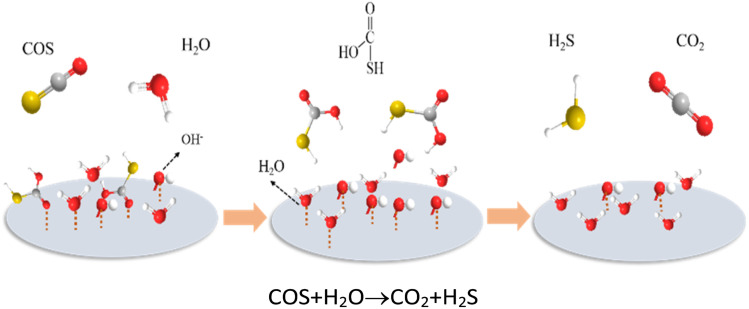
Schematic diagram of the hydrolysis mechanism of COS on MA-LDHs-*x* (this figure has been adapted/reproduced from ref. 58 with permission from Royal Soceity of Chemistry, copyright 2019).

As shown in Fig. 9, Mi *et al.*^58^ used MgAl layered double hydroxide (LDH) nanosheets synthesized by a solid mechanochemical method to separate COS. The study pointed out that in the presence of water vapor, COS can pass through ionic dipoles. The sub interaction is adsorbed on the hydroxyl group to form a COS–OH intermediate, which is then reacted with H_2_O to generate H_2_S and CO_2_ for the purpose of separation.

**Fig. 10:**
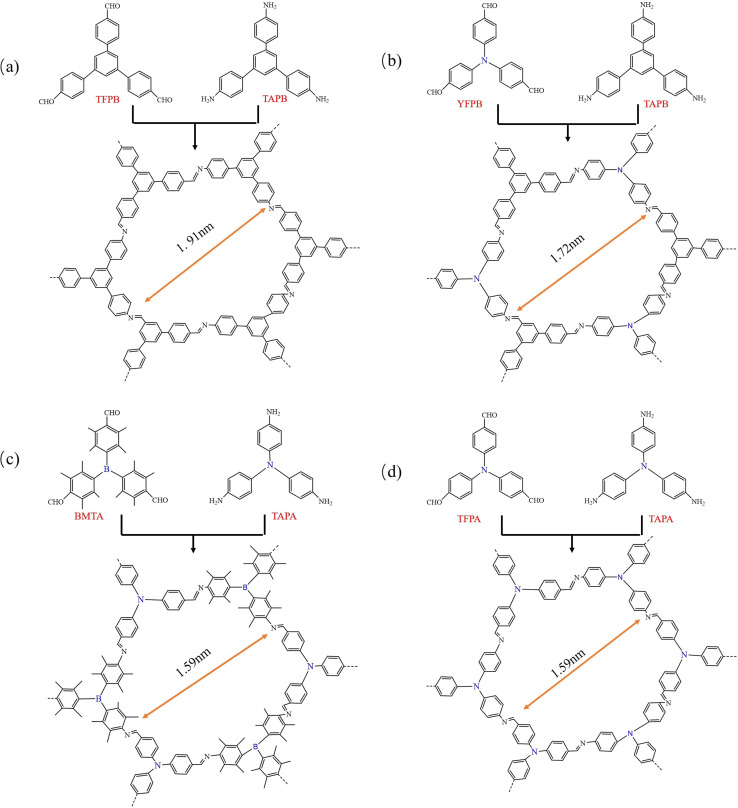
Synthetic scheme for four imine-linked hexagonal COFs (a–d) varying with the number of triarylamine groups (this figure has been adapted/reproduced from ref. 101 with permission from Royal Soceity of Chemistry, copyright 2017).

**Fig. 11:**
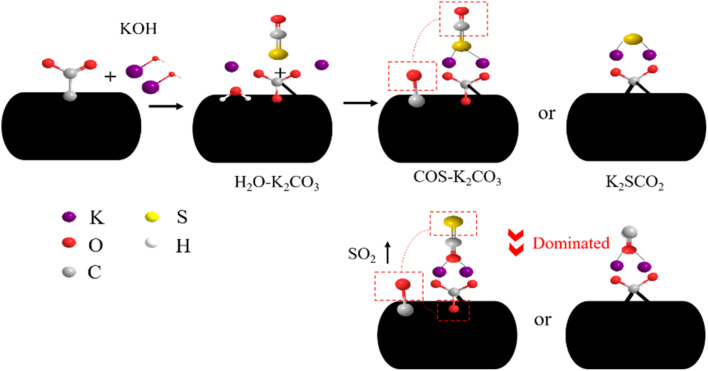
Diagram of the adsorption mechanism of COS on the surface of AC–K adsorbents.

The KOH modified walnut shell biochar catalyst was used to remove COS, and the surface properties of the catalyst were analyzed by the CO_2_-TPD method.^100^ COS and CO_2_ have similar molecular orbitals and are both Lewis acids, so the adsorption mechanism is similar. As shown in Fig. 10, Zhai *et al.*^101^ compared the adsorption capacity of the number of triaromatic amine groups on CO_2_ and found that the adsorption capacity of CO_2_ increased with the increase of the number of triaromatic amine groups, and analyzed that the high adsorption capacity of nitrogenous COFs was due to the existence of nitrogen Lewis bases in the nitrogenous COFs bond and the small pore size. As shown in Fig. 11, Kim *et al.*^75^ loaded K onto AC by the impregnation method, and the maximum adsorption capacity was increased to 55.20 mg COS per g. –OH increases the surface alkalinity of the adsorbent and promotes the oxidation of COS, and K^+^ reacts with –COO to form K_2_CO_3_ and COS. At the same time, based on the theory of soft and hard acid–base (HSAB), K^+^ soft base will more easily combine with S in COS to form K_2_SCO_2_, to achieve the purpose of separation.

#### 3.2.3 Sulfur–metal (S–M) direct interaction

**Fig. 12:**
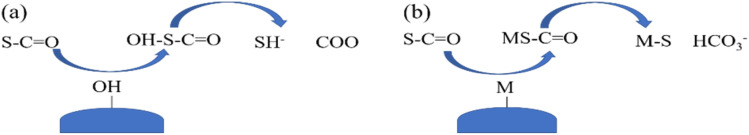
Two adsorption mechanisms of COS on the surface of adsorbent ((a) to form carbon dioxide and hydrogen sulfide; (b) forming corresponding metal sulfides).

The lone pair electrons of the sulfur atom in the organosulfur compound are donated to the metal atom in the adsorbent to directly form the sulfur–metal σ bond.^102–104^ At present, the most studied modification method is to add metal oxides to the original adsorbent. Not only can this method increase the content of adsorbed oxygen on the surface of the adsorbent, but introduced metal ions will also form S–M interactions with COS, thereby achieving the purpose of desulfurization.^71,105^ The researchers loaded the transition metal onto the Y-type molecular sieve and evaluated its desulfurization performance. The molecular sieve modified with metal Ce was found to have an adsorption capacity of 10 mg g^−1^. Because Ce ions are f-block elements with high positive charges, the S–M interaction is greater than the formation of -complexes.^106^ As shown in Fig. 12, Jiang *et al.*^107^ studied the adsorption behavior of COS on the surface of a molecular sieve adsorbent by *in situ* infrared, and found that its adsorption mechanism on the adsorbent surface is mainly divided into two categories. The main adsorption sites in Fig. 12(a) are surface bridge hydroxyl groups (Si–OH–Al) and silyl hydroxyl groups (Si–OH); Fig. 12(b) removes COS on the adsorbent based on the M–S interaction of metal cations with S.

**Fig. 13:**
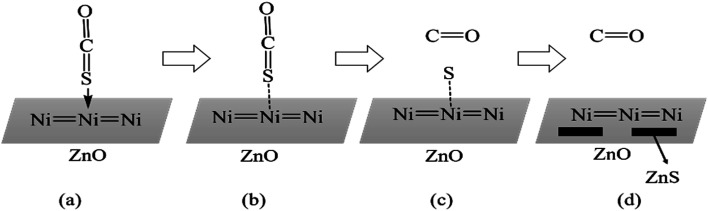
Desulfurization mechanism of reduced Ni and COS reactions (this figure has been adapted/reproduced from ref. 108 with permission from Elsevier, copyright 2015).

The adsorption process of COS was simulated as shown in Fig. 13. Zhou *et al.*^108^ compared the XRD patterns of NiO/ZnO–Al_2_O_3_ adsorbents before and after the reaction and found that the adsorbent after adsorption showed the characteristic diffraction peaks of ZnS, and the specific surface area and pore volume were significantly reduced, which proved that COS was removed as the form of ZnS, which blocked part of the pore. NiO on the adsorbent surface was reduced to Ni atoms, which combined with S to form an intermediate transition state (Fig. 13(b)). Because the C=S bond is easily broken to form various forms of nickel sulfide, which then reacts with the ZnO carrier to form ZnS, achieving the goal of separation and regeneration *via* high-temperature calcination.

In the case of HKUST-1, the copper atom in the frame is coordinated by the tricarboxylate (BTC) linker and four oxygen of the water molecule to form a face-centered cubic crystal, which contains large square pores (9.9 A), and then a frame of empty Cu^2+^ species is formed *via* a dehydration process. The coordination unsaturated Cu^2+^ cations constitute a surface ion pair, which plays a role in the adsorption process.^109^ Song *et al.*^98^ supported Fe species on the modified walnut shell biochar catalyst (abbreviation: KOH/WSB) and found that the medium-strength Fe–S binding energy (−4.5 eV) had the best performance. The iron ion shell was an unfilled structure, which gives it more nuclear charge, and this structure facilitates the formation of coordination compounds during the reaction. Coordination compounds, as intermediate products, can provide coordination catalysis and the corresponding surface reactions, thereby promoting catalytic reactions.

## 4 Conclusion and outlook

Under the background of “ultra-low emissions” in the iron and steel industry, the implementation of BFG's sulfide control can greatly reduce the overall SO_2_ emissions. Therefore, BFG desulfurization is imperative. The adsorption method to remove carbonyl sulfide has the advantages of convenient operation, no by-products, rich adsorbent, *etc.*, and its adsorption mechanism is divided into two types: physical adsorption and chemical adsorption. The former is the use of intermolecular forces to achieve the removal effect, such as the selective catalytic effect of molecular sieves. The latter chemical adsorption is based on the formation of a new bond between the active site on the adsorbent and the carbonyl sulfide, and the old fracture completes the adsorption, such as π complexation, S–M, and σ bonding. In the adsorption process, the solitary pair electrons of sulfur atoms in organic sulfur and the adsorbent containing π electrons combine with the metal atoms in the adsorbent to form sulfur–metal σ bonds and π–σ bonds, so that simple substances S and sulfates are formed on the surface of the adsorbent to achieve the purpose of removal. The core technical difficulty of BFG desulfurization is that the sulfur capacity and selectivity of the adsorbent cannot meet the requirements of industrial applications under complex working conditions. Therefore, industrial adsorbents of BFG desulfurization that can effectively remove a variety of sulfides and have economic competitiveness under the practical working conditions have become the research hotspot. This paper introduces the industrial application of BFG desulfurization and the research progress of COS. Combined with the core requirements of BFG desulfurization, this paper points out the research direction of COS adsorption materials and adsorption separation process in the future.

## Conflicts of interest

There are no conflicts to declare.

## Supplementary Material
